# Cerebrovascular and Alzheimer’s disease biomarkers in dementia with Lewy bodies and other dementias

**DOI:** 10.1093/braincomms/fcae290

**Published:** 2024-08-28

**Authors:** Anna Rennie, Urban Ekman, Sara Shams, Lina Rydén, Jessica Samuelsson, Anna Zettergren, Silke Kern, Ketil Oppedal, Frédéric Blanc, Jakub Hort, Sara Garcia-Ptacek, Angelo Antonini, Afina W Lemstra, Alessandro Padovani, Milica Gregoric Kramberger, Irena Rektorová, Zuzana Walker, Jón Snædal, Matteo Pardini, John-Paul Taylor, Laura Bonanni, Tobias Granberg, Dag Aarsland, Ingmar Skoog, Lars-Olof Wahlund, Miia Kivipelto, Eric Westman, Daniel Ferreira

**Affiliations:** Division of Clinical Geriatrics, Center for Alzheimer Research, Department of Neurobiology, Care Sciences and Society, Karolinska Institutet, 171 77 Stockholm, Sweden; Division of Clinical Geriatrics, Center for Alzheimer Research, Department of Neurobiology, Care Sciences and Society, Karolinska Institutet, 171 77 Stockholm, Sweden; Medical Unit, Allied Health Professionals Women´s Health, Karolinska University Hospital, 171 76 Stockholm, Sweden; Department of Radiology, Karolinska University Hospital, 171 76 Stockholm, Sweden; Department of Clinical Neuroscience, Karolinska Institutet, 171 77 Stockholm, Sweden; Department of Radiology, Stanford University, Stanford, 94305-5105 CA, USA; Neuropsychiatric Epidemiology Unit, Department of Psychiatry and Neurochemistry, Institute of Neuroscience and Physiology, Sahlgrenska Academy at the University of Gothenburg, 431 41 Mölndal, Sweden; Centre for Ageing and Health (AgeCap), University of Gothenburg, 413 46 Gothenburg, Sweden; Neuropsychiatric Epidemiology Unit, Department of Psychiatry and Neurochemistry, Institute of Neuroscience and Physiology, Sahlgrenska Academy at the University of Gothenburg, 431 41 Mölndal, Sweden; Centre for Ageing and Health (AgeCap), University of Gothenburg, 413 46 Gothenburg, Sweden; Neuropsychiatric Epidemiology Unit, Department of Psychiatry and Neurochemistry, Institute of Neuroscience and Physiology, Sahlgrenska Academy at the University of Gothenburg, 431 41 Mölndal, Sweden; Centre for Ageing and Health (AgeCap), University of Gothenburg, 413 46 Gothenburg, Sweden; Neuropsychiatric Epidemiology Unit, Department of Psychiatry and Neurochemistry, Institute of Neuroscience and Physiology, Sahlgrenska Academy at the University of Gothenburg, 431 41 Mölndal, Sweden; Centre for Ageing and Health (AgeCap), University of Gothenburg, 413 46 Gothenburg, Sweden; Psychiatry, Cognition and Old Age Psychiatry Clinic, Region Västra Götaland, Sahlgrenska University Hospital, 431 41 Gothenburg, Sweden; Center for Age-Related Medicine, Stavanger University Hospital, 4011 Stavanger, Norway; Stavanger Medical Imaging Laboratory (SMIL), Department of Radiology, Stavanger University Hospital, 4016 Stavanger, Norway; The Norwegian Centre for Movement Disorders, Stavanger University Hospital, 4011 Stavanger, Norway; Day Hospital of Geriatrics, Memory Resource and Research Centre (CM2R) of Strasbourg, Department of Geriatrics, Hopitaux Universitaires de Strasbourg, 67098 Strasbourg, France; ICube Laboratory and Federation de Medecine Translationnelle de Strasbourg (FMTS), University of Strasbourg and French National Centre for Scientific Research (CNRS), Team Imagerie Multimodale Integrative en Sante (IMIS)/ICONE, 67000 Strasbourg, France; Memory Clinic, Department of Neurology, Second Faculty of Medicine, Charles University and Motol University Hospital, 150 06 Prague, Czech Republic; Division of Clinical Geriatrics, Center for Alzheimer Research, Department of Neurobiology, Care Sciences and Society, Karolinska Institutet, 171 77 Stockholm, Sweden; Aging and Inflammation Theme, Karolinska University Hospital, 171 76 Stockholm, Sweden; Parkinson and Movement Disorders Unit, Study Center on Neurodegeneration (CESNE), 35129 Padova, Italy; Alzheimer Center Amsterdam, Neurology, Vrije Universiteit Amsterdam, Amsterdam UMC location Vumc, 1081 HV Amsterdam, The Netherlands; Amsterdam Neuroscience, Neurodegeneration, Vrije Universiteit Amsterdam, Amsterdam UMC location Vumc, 1081 HV Amsterdam, The Netherlands; Neurology Unit, Department of Clinical and Experimental Sciences (DSCS), University of Brescia, 25123 Brescia, Italy; Division of Clinical Geriatrics, Center for Alzheimer Research, Department of Neurobiology, Care Sciences and Society, Karolinska Institutet, 171 77 Stockholm, Sweden; Department of Neurology, University Medical Center, 1000 Ljubljana, Slovenia; Medical Faculty, University of Ljubljana, 1000 Ljubljana, Slovenia; Applied Neuroscience Research Group, CEITEC, Masaryk University, 625 00 Brno, Czech Republic; Division of Psychiatry, University College London, W1T 7NF London, UK; St Margaret's Hospital, Essex Partnership University NHS Foundation Trust, CM16 6TN Essex, UK; Memory Clinic, Landspitali, 105 Reykjavik, Iceland; Department of Neurology, IRCCS Ospedale Policlinico San Martino, 16132 Genoa, Italy; Department of Neuroscience, Rehabilitation, Ophthalmology, Genetics, Maternal and Child Health (DINOGMI), University of Genoa, 16132 Genoa, Italy; Translational and Clinical Research Institute, Faculty of Medical Sciences, Newcastle University, NE1 7RU Newcastle upon Tyne, UK; Department of Medicine, Aging Sciences University G. d'Annunzio of Chieti-Pescara Chieti, 66100 Chieti, Italy; Department of Radiology, Karolinska University Hospital, 171 76 Stockholm, Sweden; Department of Clinical Neuroscience, Karolinska Institutet, 171 77 Stockholm, Sweden; Center for Age-Related Medicine, Stavanger University Hospital, 4011 Stavanger, Norway; Department of Old Age Psychiatry, Institute of Psychiatry, Psychology & Neuroscience (IoPPN), King's College London, SE5 8AF London, UK; Neuropsychiatric Epidemiology Unit, Department of Psychiatry and Neurochemistry, Institute of Neuroscience and Physiology, Sahlgrenska Academy at the University of Gothenburg, 431 41 Mölndal, Sweden; Centre for Ageing and Health (AgeCap), University of Gothenburg, 413 46 Gothenburg, Sweden; Psychiatry, Cognition and Old Age Psychiatry Clinic, Region Västra Götaland, Sahlgrenska University Hospital, 431 41 Gothenburg, Sweden; Division of Clinical Geriatrics, Center for Alzheimer Research, Department of Neurobiology, Care Sciences and Society, Karolinska Institutet, 171 77 Stockholm, Sweden; Division of Clinical Geriatrics, Center for Alzheimer Research, Department of Neurobiology, Care Sciences and Society, Karolinska Institutet, 171 77 Stockholm, Sweden; Aging and Inflammation Theme, Karolinska University Hospital, 171 76 Stockholm, Sweden; Ageing Epidemiology Research Unit, School of Public Health, Imperial College London, SW7 2AZ London, UK; Institute of Public Health and Clinical Nutrition, University of Eastern Finland, 70211 Kuopio, Finland; Division of Clinical Geriatrics, Center for Alzheimer Research, Department of Neurobiology, Care Sciences and Society, Karolinska Institutet, 171 77 Stockholm, Sweden; Department of Neuroimaging, Centre for Neuroimaging Sciences, Institute of Psychiatry, Psychology and Neuroscience, King's College London, SE5 8AF London, UK; Division of Clinical Geriatrics, Center for Alzheimer Research, Department of Neurobiology, Care Sciences and Society, Karolinska Institutet, 171 77 Stockholm, Sweden; Facultad de Ciencias de la Salud, Universidad Fernando Pessoa Canarias, 35016 Las Palmas, España

**Keywords:** imaging, naturalistic cohort, atrophy, multi-cohort

## Abstract

Co-pathologies are common in dementia with Lewy bodies and other dementia disorders. We investigated cerebrovascular and Alzheimer’s disease co-pathologies in patients with dementia with Lewy bodies in comparison with patients with mild cognitive impairment, Alzheimer’s disease, mixed dementia, vascular dementia or Parkinson’s disease with dementia and cognitively unimpaired participants. We assessed the association of biomarkers of cerebrovascular and Alzheimer’s disease co-pathologies with medial temporal atrophy and global cognitive performance. Additionally, we evaluated whether the findings were specific to dementia with Lewy bodies. We gathered a multi-cohort dataset of 4549 participants (dementia with Lewy bodies = 331, cognitively unimpaired = 1505, mild cognitive impairment = 1489, Alzheimer’s disease = 708, mixed dementia = 268, vascular dementia = 148, Parkinson’s disease with dementia = 120) from the MemClin Study, Karolinska Imaging in Dementia Study, Gothenburg H70 Birth Cohort Studies and the European DLB Consortium. Cerebrovascular co-pathology was assessed with visual ratings of white matter hyperintensities using the Fazekas scale through structural imaging. Alzheimer’s disease biomarkers of β-amyloid and phosphorylated tau were assessed in the cerebrospinal fluid for a subsample (*N* = 2191). Medial temporal atrophy was assessed with visual ratings and global cognition with the mini-mental state examination. Differences and associations were assessed through regression models, including interaction terms. In dementia with Lewy bodies, 43% had a high white matter hyperintensity load, which was significantly higher than that in cognitively unimpaired (14%), mild cognitive impairment (26%) and Alzheimer’s disease (27%), but lower than that in vascular dementia (62%). In dementia with Lewy bodies, white matter hyperintensities were associated with medial temporal atrophy, and the interaction term showed that this association was stronger than that in cognitively unimpaired and mixed dementia. However, the association between white matter hyperintensities and medial temporal atrophy was non-significant when β-amyloid was included in the model. Instead, β-amyloid predicted medial temporal atrophy in dementia with Lewy bodies, in contrast to the findings in mild cognitive impairment where medial temporal atrophy scores were independent of β-amyloid. Dementia with Lewy bodies had the lowest performance on global cognition, but this was not associated with white matter hyperintensities. In Alzheimer’s disease, global cognitive performance was lower in patients with more white matter hyperintensities. We conclude that white matter hyperintensities are common in dementia with Lewy bodies and are associated with more atrophy in medial temporal lobes, but this association depended on β-amyloid-related pathology in our cohort. The associations between biomarkers were overall stronger in dementia with Lewy bodies than in some of the other diagnostic groups.

## Teaser

Rennie *et al*. reported that white matter hyperintensities, a neuroimaging marker of cerebrovascular disease, are common in dementia with Lewy bodies. White matter hyperintensities were associated with atrophy in the medial temporal lobe. The association of white matter hyperintensities with other biomarkers and cognitive performance was different across diagnostic groups.

## Graphical Abstract

**Graphical abstract:**
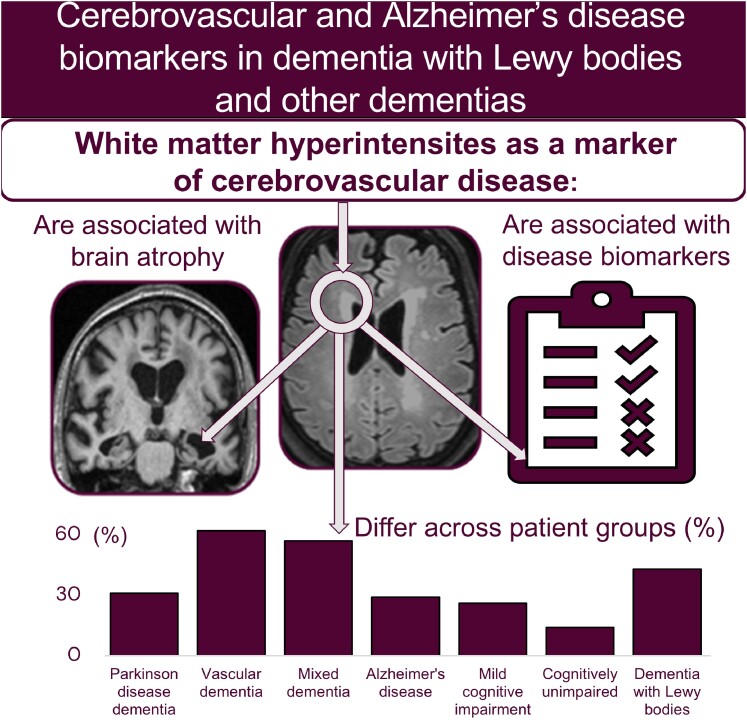

## Introduction

Dementia with Lewy bodies is a clinically heterogeneous disorder characterized by variability in symptoms primarily associated with Lewy body pathology.^1^ However, some of this variability in symptoms has been associated with cerebrovascular and Alzheimer’s disease co-pathologies.^2,3^ This is not exclusive to dementia with Lewy bodies, as co-pathologies are also common in other dementias and contribute to their patterns of atrophy, clinical characteristics and cognitive signatures.^3-7^ Hence, it is essential to investigate these associations and contributions within dementia with Lewy bodies but also in comparison with other dementias.

Co-pathologies can be assessed *in vivo* through biomarkers. Cerebrovascular co-pathology is commonly assessed through white matter hyperintensities (WMHs) on magnetic resonance imaging (MRI).^8^ In dementia with Lewy bodies, the frequency and effect of WMHs are debated. A recent review suggested that patients with dementia with Lewy bodies often have more WMHs than healthy controls and patients with Alzheimer’s disease, whilst their clinical contribution is not fully established.^2^ Two recent studies reported an association between more WMHs and poorer cognition in dementia with Lewy bodies.^9,10^ Moreover, WMHs have been widely investigated in populations other than dementia with Lewy bodies. For example, WMHs are associated with an increased risk for all-cause dementia, including Alzheimer’s disease and vascular dementia,^11^ and with poorer cognition.^8^ Regarding Alzheimer’s disease co-pathology, β-amyloid (Aβ) and tau neurofibrillary tangles can be assessed in the cerebrospinal fluid (CSF) or on positron emission tomography. Alzheimer’s disease biomarkers are positive in around 50% of patients with dementia with Lewy bodies and are associated with worse cognition.^3,12^ However, to our knowledge, the association between WMHs and Aβ has not been studied in dementia with Lewy bodies before. In non-dementia with Lewy bodies populations, the review by Roseborough *et al.*^13^ suggested that the association between WMHs and Aβ remains unclear, but WMHs may influence Aβ accumulation over time.

For these reasons, distinguishing dementia with Lewy bodies from other dementias can be challenging. In the clinical setting, the relative sparing of the medial temporal lobe is a supportive biomarker for the diagnosis of dementia with Lewy bodies.^1^ Medial temporal atrophy (MTA) is commonly assessed in clinical practice with MRI or computer tomography (CT).^14^ Although MTA can be supportive in distinguishing between dementia with Lewy bodies and Alzheimer’s disease patients,^15,16^ dementia with Lewy bodies patients with Alzheimer’s disease co-pathology have more MTA than dementia with Lewy bodies patients without Alzheimer’s disease co-pathology.^17,18^ However, atrophy has seldom been investigated together with biomarkers of cerebrovascular and Alzheimer’s disease co-pathologies.^19^ Therefore, their associations with each other and whether those associations are specific to dementia with Lewy bodies remains largely unknown.

The first aim of our current study was to investigate the frequency of WMHs in patients with dementia with Lewy bodies in comparison to groups of patients with other dementias, mild cognitive impairment and cognitively unimpaired participants and to elucidate if WMHs are associated with MTA, Aβ and phosphorylated tau (p-tau) biomarkers of Alzheimer’s disease and cognitive performance. The second aim was to determine if these associations were specific to dementia with Lewy bodies by testing for statistical interactions with the other diagnostic groups. In line with previous literature, we hypothesized that patients with dementia with Lewy bodies would have a higher frequency of WMHs compared to the groups of cognitively unimpaired, mild cognitive impairment and Alzheimer’s disease participants, a lower frequency compared to patients with vascular dementia and mixed dementia (i.e. Alzheimer’s disease plus vascular dementia), and a similar frequency compared to patients with Parkinson’s disease with dementia. We further hypothesized that WMHs would be associated with more MTA, Aβ positivity and worse cognitive performance in patients with dementia with Lewy bodies. Finally, we hypothesized that WMHs and Alzheimer’s disease biomarkers would independently contribute to MTA in patients with dementia with Lewy bodies. Regarding the specificity of the associations, we hypothesized that some associations found in dementia with Lewy bodies could be shared with Alzheimer’s disease and with groups with vascular aetiology such as vascular dementia and mixed dementia. To address these aims and hypotheses, we assembled a large multi-cohort dataset of 4549 individuals including multiple diagnostic groups.

## Materials and methods

### Participants

Participants were enrolled from four large cohorts: the European DLB Consortium (E-DLB, *N* = 546),^20^ the Gothenburg H70 Birth Cohort Studies (H70*, N* = 774),^21^ the Karolinska Imaging Dementia Study (KIDS, *N* = 1312)^22^ and the MemClin Study (*N* = 1917),^23^ for a total of 4549 individuals as follows: dementia with Lewy bodies = 331, cognitively unimpaired = 1505, mild cognitive impairment = 1489, Alzheimer’s disease = 708, mixed dementia = 268, vascular dementia = 148 and Parkinson’s disease with dementia = 120 (Table 1 and Supplementary Table 1). We included all eligible individuals with scores available on the Fazekas rating scale for WMHs (see the next section), who were at least 45 years old and received one of the diagnoses of interest in this study or were cognitively unimpaired.

**Table 1:** Cohort characteristics

	Dementia with Lewy bodies	Cognitively unimpaired	Mild cognitive impairment	Alzheimer’s disease	Mixed dementia	Vascular dementia	Parkinson’s disease with dementia	Entire cohort
*N*	311	1505	1489	708	268	148	120	4549
Age (years)	73.4 (8.1)	67.5 (7.9)	72.9 (9.7)	72.3 (9.1)	76.2 (8.1)	74.0 (10.3)	70.6 (7.4)	71.2 (9.3)
Women, *n* (%)	84 (38)	880 (59)	712 (48)	419 (59)	126 (47)	62 (42)	32 (27)	2350 (52)
Education (years)	11.0 (4.0)	13.5 (4.0)	12.9 (6.6)	11.9 (3.6)	12.0 (3.6)	11.2 (3.3)	10.2 (4.3)	12.6 (3.9)
MMSE, total score	22.7 (4.0)	28.6 (2.1)	26.9 (2.6)	23.2 (4.0)	23.41 (4.8)	23.4 (4.5)	23.7 (4.0)	26.2 (3.9)
WMHs, high score, *n* (%)	135 (43)	215 (14)	388 (26)	188 (27)	136 (51)	92 (62)	37 (31)	1191 (26)
MTA, high score, *n* (%)	125 (48)	303 (21)	445 (41)	274 (61)	166 (66)	80 (57)	39 (45)	1441 (41)
Aβ, positive, *n* (%)	28 (33)	108 (15)	228 (29)	246 (68)	95(61)	15 (24)	-	726 (33)
p-tau, positive, *n* (%)	31 (37)	59 (8)	201 (27)	209 (58)	68 (44)	-	-	567 (26)

Data are reported as mean (SD), otherwise, count (%) when frequencies are reported. Education *N* = 2881, MMSE *N* = 2724, MTA *N* = 3508, Aβ and p-tau analysis *N* = 2191. Analyses for Aβ and p-tau were only performed in groups with sufficient data (≥8 cases per cell): Parkinson’s disease with dementia had to be excluded from the analysis of both Aβ and p-tau, and the vascular dementia group had to be excluded from the analysis of p-tau. For Parkinson’s disease with dementia, 28 individuals had available data, with only six individuals having a positive Aβ biomarker and 0 a positive p-tau biomarker. For vascular dementia, 61 individuals had available data for p-tau, with only three individuals having a positive p-tau biomarker.

Aβ, β-amyloid; MMSE, mini-mental state examination; MTA, medial temporal atrophy; p-tau, phosphorylated tau; WMHs, white matter hyperintensities.

Diagnostic procedures for patient groups and cognitively unimpaired were comparable across the four cohorts and are explained in the original publications^20-23^ and detailed in Supplementary Table 2. Mixed dementia in this study refers to Alzheimer’s disease plus vascular dementia as well as unspecified Alzheimer’s disease and is included because it is a common diagnosis in the clinical setting as well as a common diagnostic group in the included cohorts as International Statistical Classification of Diseases (ICD) 10 codes F00.2 and F00.9. Global cognition was assessed with the mini-mental state examination (MMSE).^24^ Neuroimaging, CSF biomarkers and MMSE were used in the diagnostic workup. However, neuroimaging was only used in an unstructured manner for radiological assessment; cognitive impairment and establishment of cognitive profiles were done using extensive neuropsychological protocols above and beyond MMSE; and CSF biomarkers were supportive only for the dementia groups and were available for a subsample (*N* = 2191). The final diagnosis was thus based on clinical judgement. Although neuroimaging, CSF biomarkers and MMSE are the main variables of interest in this study, the risk for circularity is low and, if any, it would only affect one part of aim one, i.e. investigate the frequency of WMHs in dementia with Lewy bodies in comparison with other diagnostic groups, but it should not affect the hypotheses related to associations between measures.

Our present study received ethical approval from the Swedish Ethics Review Authority, and in addition, the included cohorts had their own ethical approvals. This research follows the Declaration of Helsinki.

### Neuroimaging measures of white matter hyperintensities and medial temporal atrophy

Protocols for the acquisition of neuroimaging data in each cohort are described elsewhere.^20-23^ Although protocols are standard and largely comparable between cohorts, due to the multiple scanners involved and the clinical focus of this study we favoured clinical measures of WMH and regional atrophy instead of more advanced research-oriented quantitative measures. Thus, WMHs were assessed with the Fazekas scale,^25^ a radiological visual rating scale widely used in clinical settings as a measure of WMHs of presumed vascular origin.^8^ Fazekas scores range from 0 to 3, with a score of 0 indicating no or few punctate white matter changes, a score of 1 indicating multiple punctate changes, a score of 2 indicating white matter changes that start to become confluent and a score of 3 indicating changes that are fully confluent.^14^ Following previous publications by Joki et al.,^26^ we determined Fazekas abnormality based on a cut point of 2, which provided two groups: Fazekas 0–1 versus 2–3, with age adjustments performed in subsequent statistical analysis by including age as a covariate in the statistical models (see the statistical analysis section for a description of all the statistical models). MTA was assessed with the Scheltens scale,^27^ which is a visual rating scale that ranges from 0 to 4 and as the Fazekas scale, and it is also widely used in clinical settings.^14^ An MTA score of 0 denotes a normal width of the temporal horn and choroid fissure as well as a normal hippocampus, a score of 1 denotes that the choroid fissure is slightly expanded, whilst in scores 2–4, the enlargement of choroid fissure and temporal horn, as well as the decreased hippocampal height, are progressively more pronounced.^14^ We determined MTA abnormality using the cut points published in Ferreira *et al.*,^28^ as follows: scores ≥1.5 for individuals below 75 years of age, ≥ 2 for individuals between 75 and 84 years and ≥2.5 for individuals older than 85.

Two neuroradiologists with long-time experience in rating scans clinically together applied the Fazekas scale in E-DLB, H70 and KIDS, as well as the MTA rating in E-DLB and KIDS. The two neuroradiologists have previously shown good inter-rater agreement between them on independent ratings.^29^ Participants from H70 had MTA assessed with an artificial intelligence method trained on scores from one of our two neuroradiologists.^30^ For MemClin, we used ratings from the radiological centre performing the scan,^23^ which were performed either on MRI (*n* = 657, 34% of the participants) or on CT (*n* = 1258, 66%). Since previous studies have shown a good agreement for Fazekas and MTA scores across MRI and CT scans,^31^ CT and MRI ratings from MemClin were combined for statistical analyses in our current study. Additionally, previous studies have shown a strong correlation between hyperintensities and hypointensities on MRI.^32^ Hence, in this article we use the term WMHs, although they will appear hypointense on CT imaging for 1258 individuals from MemClin. This was done for simplicity and to better align with the current research terminology of WMHs of presumed vascular origin.^8^ Otherwise, all ratings from E-DLB (*N* = 546), H70 (*N* = 774) and KIDS (*N* = 1312) were performed on MRI scans. Imaging data was managed through theHiveDB.^33^

### Cerebrospinal fluid biomarkers of Alzheimer’s disease pathology

In a subsample, we assessed Aβ and tau pathology through CSF biomarkers of Aβ42 and p-tau 181, respectively. See Table 1 for the proportion of participants with CSF biomarkers and Supplementary Table 1 for that proportion in each cohort. We defined biomarker positivity based on centre-specific cut points to be able to combine the data from all four cohorts. Cohort-specific cut points and procedures are fully detailed elsewhere and summarized in Supplementary Table 3.^23,34-36^

### Statistical analysis

We evaluated cohort characteristics using ANOVA for age and education and the *χ*^2^ test for sex distribution. All subsequent models were adjusted for age and sex, and MMSE analyses were additionally adjusted for years of education.

We used binary logistic regressions for dichotomous outcomes (i.e. WMHs, MTA and CSF biomarkers) and multiple linear regression for continuous outcomes (i.e. MMSE). Specifically, we compared the frequency of WMHs and MTA across diagnostic groups by performing binary logistic regressions, with WMHs and MTA as outcome variables in separated models and the diagnostic group as a predictor. We also used binary logistic regression to evaluate the association of WMHs (predictor) with MTA, Aβ and p-tau (outcomes in three separate models), first in patients with dementia with Lewy bodies alone and then across all diagnostic groups. Next, we assessed Aβ and p-tau in addition to WMHs in predicting MTA, with all predictors in the same binary logistic regression model. Furthermore, we evaluated WMHs in predicting MMSE through multiple linear regression, first in patients with dementia with Lewy bodies alone and then across all diagnostic groups. Finally, to assess whether results were specific to dementia with Lewy bodies, we tested for the statistical interaction between the biomarker of interest and the diagnostic group (dementia with Lewy bodies as the reference group compared to cognitively unimpaired, mild cognitive impairment, Alzheimer’s disease, mixed dementia, vascular dementia and Parkinson’s disease with dementia). For example, for the association of WMHs with MTA, we fitted a model with an interaction term for WMHs by the diagnostic group (in addition to WMHs and the diagnostic group main effects as well as sex and age covariates as extra predictors) in predicting MTA. *Post hoc* tests after significant interaction terms were performed with the *χ*^2^ test and analysis of covariance (ANCOVA) to assess differences pairwise for dementia with Lewy bodies versus the other diagnostic groups, for categorical and continuous outcomes, respectively. Cramer *V* was used to estimate effect sizes after *χ*^2^ tests. Odds ratios are presented for the binary logistic regressions.

All statistical analyses were performed in R Studio and the alpha level was set to 0.05, with 95% confidence intervals.

## Results

### Cohort characteristics

Table 1 shows that compared to the dementia with Lewy bodies, cognitively unimpaired and Parkinson’s disease with dementia groups were significantly younger, and the mixed dementia group was older. In terms of education, the cognitively unimpaired and mild cognitive impairment groups had more years of education than the dementia with Lewy bodies group. Regarding cognition, the dementia with Lewy bodies group had the lowest MMSE scores. Compared to the dementia with Lewy bodies group, there were more women in the cognitively unimpaired, mild cognitive impairment, Alzheimer’s disease and mixed dementia groups and fewer women in the Parkinson’s disease with dementia group (Table 1). Thus, all subsequent analyses had both age and sex as covariates. Characteristics for the dementia with Lewy bodies group stratified by WMHs, MTA and Aβ biomarker status are available in Supplementary Table 4.

### White matter hyperintensities across diagnoses

**Figure 1:**
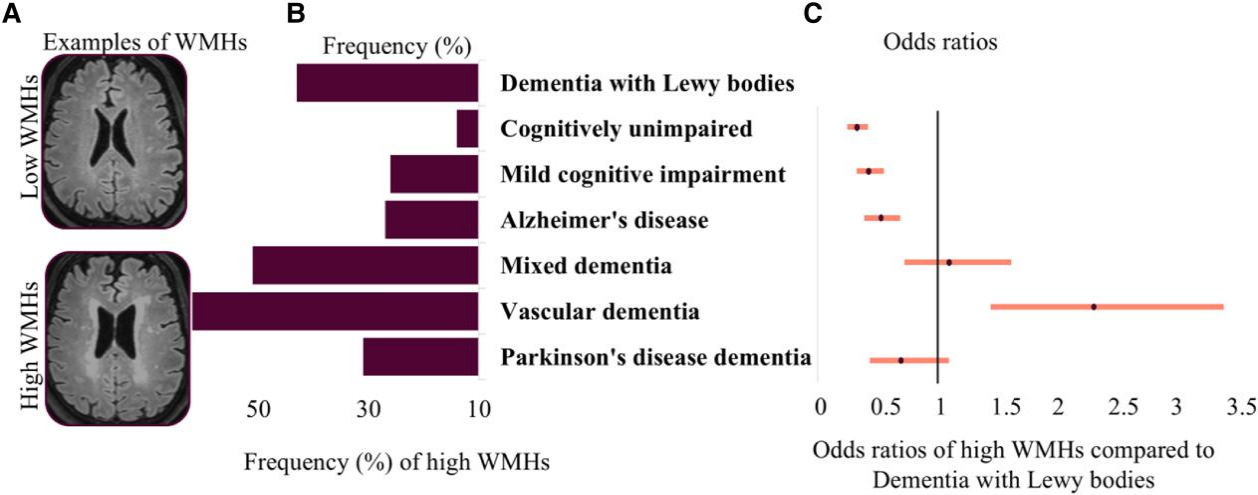
**White matter hyperintensities (WMHs) across diagnoses.** Odds ratios from a logistic regression model. Panel **A** shows examples of low and high WMHs. Panel **B** displays absolute frequencies of high WMH load (Fazekas scores 2 and 3). Panel **C** shows odds ratios based on logistic regression with the dementia with Lewy bodies group as reference. Model adjusted for age and sex. *χ*^2^(8, *N* = 4549) = 634.82, *P* < 0.001. In the logistic regression model, the WMH variable is dichotomous and was coded as a high WMH load (Fazekas scores 2 and 3) versus a low WMH load (Fazekas scores 0 and 1). Dot reflects the estimate (odds ratio) and whiskers the 95% confidence interval. Significant results compared to dementia with Lewy bodies do not cross the black line.

Results from logistic regression showed that the dementia with Lewy bodies group had significantly more WMHs than cognitively unimpaired, mild cognitive impairment and Alzheimer’s disease groups, but less WMHs than the vascular dementia group (Table 1 and Fig. 1). We observed no significant differences in WMHs between dementia with Lewy bodies and the mixed dementia and Parkinson’s disease with dementia groups.

### The association between white matter hyperintensities and medial temporal atrophy

**Figure 2:**
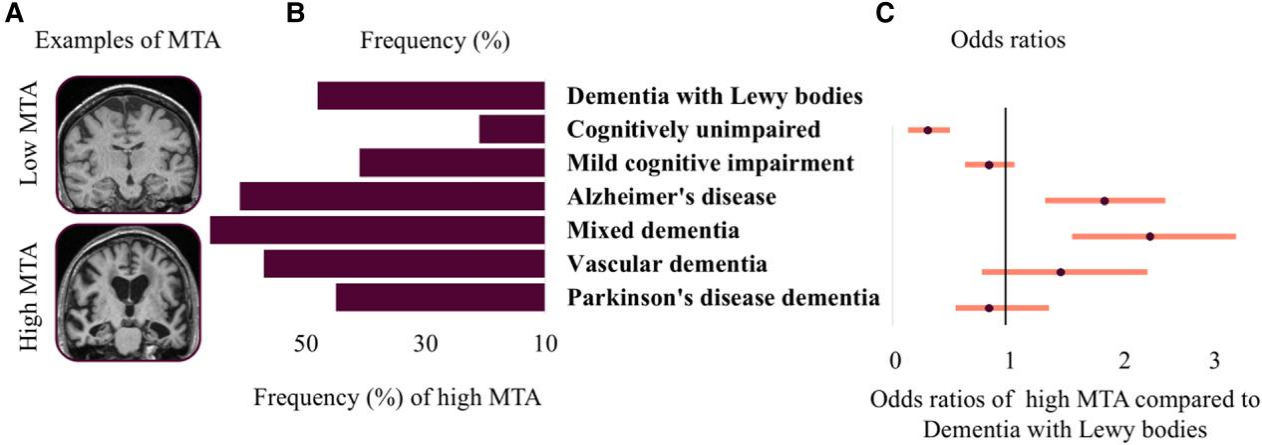
**Medial temporal atrophy (MTA) across diagnoses.** Odds ratios from a logistic regression model. Panel **A** shows examples of low and high MTA. Panel **B** displays absolute frequencies of MTA (cut-offs are age adjusted). Panel **C** shows odds ratios based on logistic regression with the dementia with Lewy bodies group as a reference. Absolute frequencies of MTA, odds ratios based on logistic regression with the dementia with Lewy bodies as a reference group. Model adjusted for age and sex, *χ*^2^(8, *N* = 3508)= 324.72, *P* < 0.001. Dot reflects the estimate and whiskers the 95% confidence interval. Significant results compared to dementia with Lewy bodies do not cross the black line.

Table 1 and Fig. 2 show the frequency of MTA across diagnoses. The dementia with Lewy bodies group had significantly more MTA than the cognitively unimpaired group but less MTA than the Alzheimer’s disease and mixed dementia groups.

**Figure 3:**
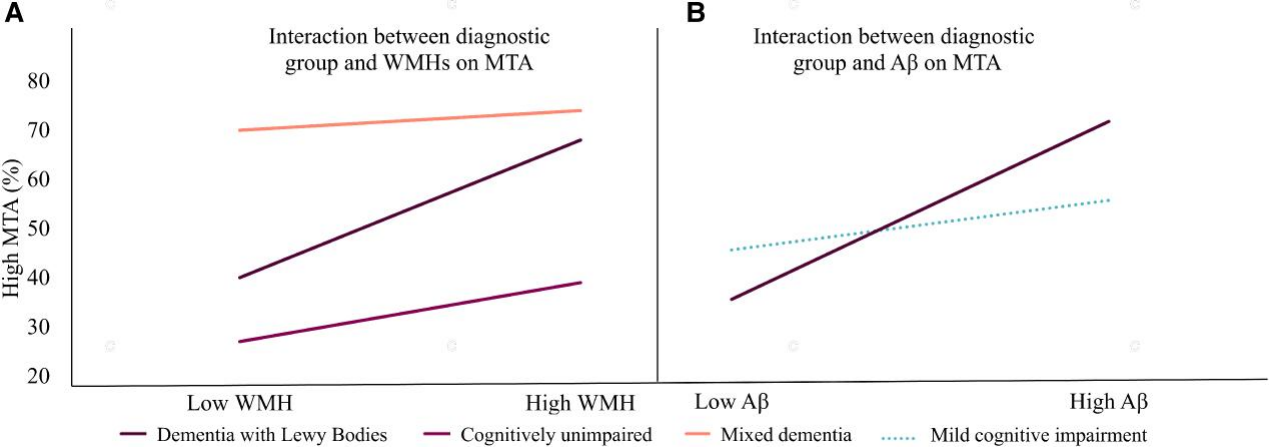
**Results from the logistic regression models in Table 3.** Interactions between biomarkers and the diagnostic group on the prediction of MTA. Only statistically significant interactions displayed. Displayed is the percent in each category with a high degree of MTA. (**A**) Interaction of the diagnostic group and WMHs on MTA; dementia with Lewy bodies in comparison with cognitively unimpaired and mixed dementia. The model is adjusted for age and sex, and main effects of the diagnostic group and WMHs are also fitted in addition to the interaction term. Omnibus statistics, *χ*^2^(15, *N* = 3508) = 414.55, *P* < 0.001. (**B**) Interaction of the diagnostic group and Aβ on MTA; dementia with Lewy bodies in comparison with mild cognitive impairment. The model is adjusted for age and sex, and main effects of the diagnostic group and MTA are also fitted in addition to the interaction term. Omnibus statistics, *χ*^2^(13, *N* = 1477) = 137.77, *P* < 0.001. MTA, medial temporal atrophy; WMH, white matter hyperintensities.

In the dementia with Lewy bodies group, more WMHs were significantly associated with more MTA (Table 2). To assess whether this association was specific to dementia with Lewy bodies, we tested for the statistical interaction between WMHs and the diagnostic group in the whole sample, with MTA as the outcome variable (Table 3 and Fig. 3A). Compared to dementia with Lewy bodies, we observed a significant interaction between WMHs and the diagnostic group for cognitively unimpaired and mixed dementia groups. Specifically, patients with dementia with Lewy bodies and more WMHs had more MTA compared to patients with dementia with Lewy bodies with less WMHs (effect size by *Cramer V* = 0.24), whilst in cognitively unimpaired and mixed dementia, MTA scores tended to be independent of WMH status (cognitively unimpaired group: *Cramer V* = 0.08; mixed dementia group: *Cramer V* = 0.01).

**Table 2:** Results for dementia with Lewy body only analyses

Model	Predictor	Estimate	95% confidence interval/*P*-value
Association of WMHs with MTA			
** ** *χ* ^2^(3, *N* = 259) = 27.701, *P* < 0.001	WMHs	1.90	**1.09–3.32**
	Age	1.36	**1.13–1.64**
	Sex	1.02	0.60–1.74
Association of WMHs, Aβ and p-tau with MTA			
** ** *χ* ^2^(5, *N* = 83) = 24.367, *P* < 0.001	WMHs	1.53	0.51–4.62
	Aβ	3.98	**1.37–12.62**
	p-tau	0.47	0.16–1.37
	Age	1.87	**1.26–2.98**
	Sex	1.05	0.27–4.21
Association of WMHs with MMSE			
** ** *F*(2, 258) = 3.335	WMHs	0.34	0.52
** **Adjusted *R*^2^ = 0.035, *P* = 0.01	Age	−0.01	0.86
	Sex	−0.24	0.64
	Education	0.22	**<0.001**

Odds ratios and 95% confidence intervals are reported for logistic regression models, and beta estimates and *P*-values are reported for multiple linear regression models. For the logistic regressions, the odds ratio for age is presented per 5 years of age. Reference groups are negative biomarkers and male sex. Significant predictors are bold.

Aβ, β-amyloid; MMSE, mini-mental state examination; MTA, medial temporal atrophy; p-tau, phosphorylated tau; WMHs, white matter hyperintensities.

**Table 3:** Results for analyses across diagnoses and interactions with the dementia with Lewy bodies group

Model	Predictor	Estimate	95% confidence interval/*P*-value
Association of WMHs with MTA—WMHs by diagnosis interaction			
*χ*^2^(15, *N* = 3508) = 414.55, *P* < 0.001	WMHs × diagnosis (cognitively unimpaired)	0.52	**0.28–0.96**
	WMHs × diagnosis (mild cognitive impairment)	0.64	0.36–1.13
	WMHs × diagnosis (Alzheimer’s disease)	0.95	0.48–1.88
	WMHs × diagnosis (mixed dementia)	0.36	**0.17–0.74**
	WMHs × diagnosis (vascular dementia)	0.71	0.30–1.71
	WMHs × diagnosis (Parkinson’s disease with dementia)	0.66	0.24–1.85
	Age	0.93	**0.88–0.97**
	Sex	0.63	**0.55–0.73**
Association of WMHs, Alzheimer’s disease biomarkers with MTA—interaction WMHs and Aβ			
*χ*^2^(13, *N* = 1477) = 137.77, *P* < 0.001	Aβ × diagnosis (cognitively unimpaired)	0.34	0.11–1.00
	Aβ × diagnosis (mild cognitive impairment)	0.32	**0.11–0.91**
	Aβ × diagnosis (Alzheimer’s disease)	0.53	0.17–1.60
	Aβ × diagnosis (mixed dementia)	0.43	0.12–1.44
	Age	0.95	0.88–1.01
	Sex	0.66	**0.53–0.823**
Association of WMHs with MMSE			
*F*(16, 2707) = 164.4, adjusted *R*^2^ 0.49, *P* < 0.001			
	WMHs × diagnosis (cognitively unimpaired)	−0.01	0.97
	WMHs × diagnosis (mild cognitive impairment)	−0.35	0.29
	WMHs × diagnosis (Alzheimer’s disease)	−0.87	**0.03**
	WMHs × diagnosis (mixed dementia)	−0.33	0.520
	WMHs × diagnosis (vascular dementia)	−0.35	0.581
	WMHs × diagnosis (Parkinson’s disease with dementia)	0.12	0.844
	Age	0.02	**0.01**
	Sex	0.03	0.77
	Education	0.11	**<0.001**

Odds ratios and 95% confidence intervals are reported for logistic regression models and beta estimates and *P*-values are reported for multiple linear regression models. For the logistic regressions, the odds ratio for age is presented per 5 years of age. Models are fitted with the main effect of the diagnostic group and biomarker of interest and interaction term of interest, e.g. the model for Association of WMHs with MTA—WMHs by diagnosis interaction additionally contain the predictors WMH and diagnosis which are not displayed in the interest of brevity. Reference groups are Dementia with Lewy bodies, negative biomarkers and male sex. Significant predictors are bold.

Aβ, β-amyloid; MMSE, mini-mental state examination; MTA, medial temporal atrophy; p-tau, phosphorylated tau; WMHs, white matter hyperintensities.

### The association between white matter hyperintensities and Alzheimer’s disease biomarkers

Alzheimer’s disease biomarker status was available for a subsample of 726 individuals who were significantly younger and had less WMHs than individuals who did not have Alzheimer’s disease biomarker testing (*P* ≤ 0.05, data not shown). In contrast, there were no statistically significant differences in MMSE, MTA or sex distribution (*P* > 0.05).

Alzheimer’s disease biomarkers across diagnoses are reported in Table 1. The dementia with Lewy bodies group had a significantly higher frequency of a positive Aβ biomarker than the cognitively unimpaired group, but a lower frequency than the Alzheimer’s disease and mixed dementia groups. In terms of p-tau, the dementia with Lewy bodies group had a higher frequency of a positive p-tau biomarker than the cognitively unimpaired and mild cognitive impairment groups, but a lower frequency than the Alzheimer’s disease group.

In dementia with Lewy bodies, a positive Aβ or p-tau biomarker was not associated with WMHs in separate models for Aβ or p-tau, respectively (*χ*^2^(3, *N* = 84) = 3.966, *P* = 0.265 and *χ*^2^(3, *N* = 84) = 3.964, *P* = 0.265). To understand if this finding was specific to dementia with Lewy bodies, we tested for the statistical interaction between WMHs and the diagnostic group, with Aβ or p-tau as outcome variables. We did not find any significant interaction between WMHs and the diagnostic group (*P* > 0.05).

### White matter hyperintensities, Alzheimer’s disease biomarkers and medial temporal atrophy

We then evaluated WMHs and Alzheimer’s disease biomarkers jointly in the prediction of MTA. For dementia with Lewy bodies, WMHs were no longer significantly associated with more MTA when Aβ and p-tau were included in the model, and only Aβ significantly predicted MTA in the presence of WMHs and p-tau (Table 2). To assess whether this finding was specific to dementia with Lewy bodies, we tested for the statistical interaction between Aβ and the diagnostic group in predicting MTA, retaining WMHs and p-tau in the model (Table 3 and Fig. 3B). We observed a significant interaction between dementia with Lewy bodies and the mild cognitive impairment group. Specifically, patients with dementia with Lewy bodies and a positive Aβ biomarker had significantly more MTA than patients with dementia with Lewy bodies and a negative Aβ biomarker (*P* > 0.05), whilst in mild cognitive impairment, MTA scores were independent of Aβ status (*P*≤0.05).

### The association between white matter hyperintensities and mini-mental state examination

**Figure 4:**
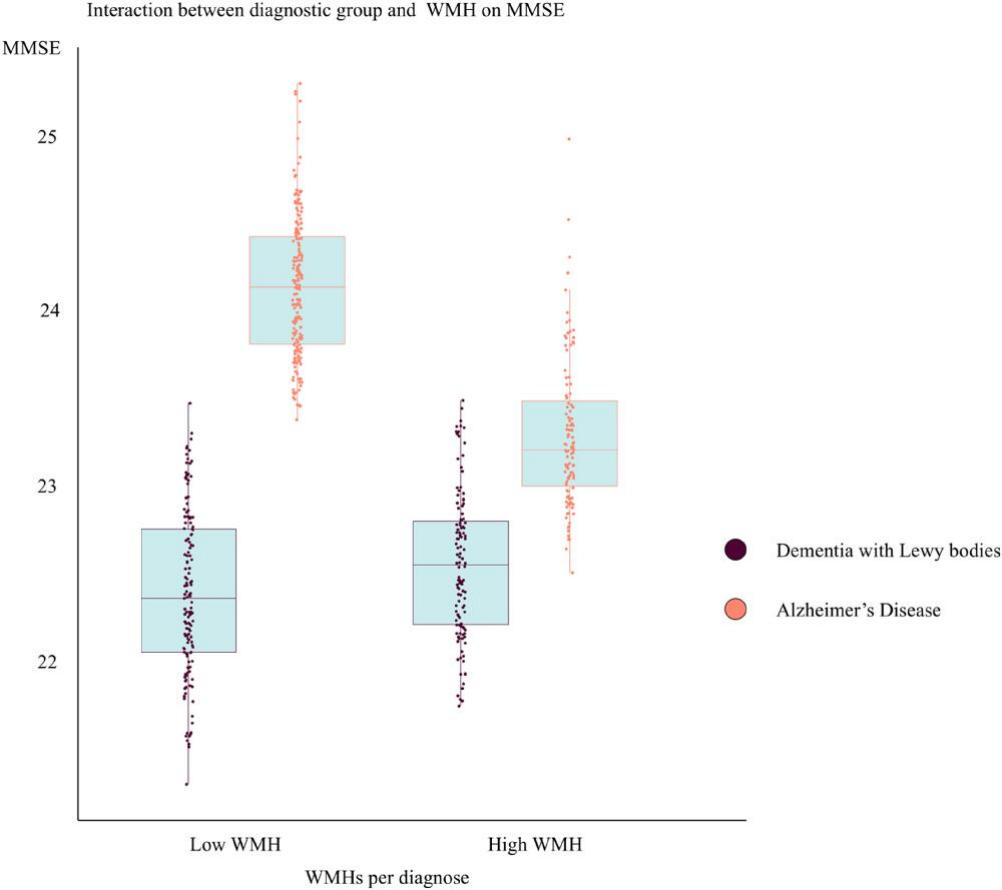
**Results from the linear regression models in Table 3.** Interaction between WMHs and diagnosis on MMSE. Only the statistically significant interaction displayed with fitted values of individual observations. Dementia with Lewy bodies in comparison to Alzheimer’s disease. The model is adjusted for age, sex and education, and main effects of diagnosis and WMHs are also fitted in addition to the interaction term. Omnibus statistics, *F*(16, 2707) = 164.4, adjusted *R*^2^ = 0.49, *P* < 0.001.

We conducted a multiple linear regression with WMHs as the predictor and MMSE as the outcome variable. In the dementia with Lewy bodies group, WMHs were not significantly associated with MMSE scores (Table 2). To understand if this finding was specific to dementia with Lewy bodies, we tested for the statistical interaction between WMHs and the diagnostic group, with MMSE as the outcome variable (Table 3 and Fig. 4). We observed a significant interaction, where compared to the dementia with Lewy bodies group, the Alzheimer’s disease group showed a significant association between WMHs and MMSE. Specifically, in dementia with Lewy bodies, MMSE performance was independent of WMHs (*P* > 0.05), whilst in the Alzheimer’s disease group, patients with more WMHs performed worse in MMSE than those with less WMHs (*P*≤0.05).

## Discussion

We investigated WMHs in relation to MTA, Alzheimer’s disease biomarkers and cognition in dementia with Lewy bodies as well in comparison with other dementias, mild cognitive impairment and being cognitively unimpaired. We first compared WMHs across diagnoses, showing that the dementia with Lewy bodies group had more WMHs than the cognitively unimpaired participants, the patients with mild cognitive impairment and Alzheimer’s disease, whilst they had less WMHs than the group with vascular dementia. We then evaluated the association of WMHs with MTA, Alzheimer’s disease biomarkers and cognition in dementia with Lewy bodies as well as in comparison with the other diagnostic groups through statistical interactions. Although several of the associations found in dementia with Lewy bodies were shared with the other diagnostic groups, we also observed some specific findings. In dementia with Lewy bodies, the association between WMHs and MTA was stronger than among the cognitively unimpaired and mixed dementia groups. Similarly, the association between Aβ and MTA was stronger in the group with dementia with Lewy bodies than in the mild cognitive impairment group. Finally, the association between WMHs and cognition was weaker in dementia with Lewy bodies than in Alzheimer’s disease group.

Dementia with Lewy bodies patients had more WMHs than the cognitively unimpaired participants and mild cognitive impairment or Alzheimer’s disease patients, whilst they had less WMHs than patients with vascular dementia. We are aware of only one previous study that compared WMHs across multiple diagnostic groups, including dementia with Lewy bodies.^37^ The authors found that patients with dementia with Lewy bodies had more WMHs than cognitively unimpaired participants and less WMHs than patients with vascular dementia, in line with our findings. However, Koikkalainen *et al.*^37^ did not find any statistically significant differences in WMHs between dementia with Lewy bodies and Alzheimer’s disease groups. Compared to our sample, Koikkalainen *et al.*^37^ had a younger sample with less WMHs overall, which could explain the different results. Moreover, although we qualitatively observed more WMHs in dementia with Lewy bodies (43%) than in Parkinson’s disease with dementia (30%), this difference did not reach statistical significance in our cohort (*P* = 0.13). The recent larger study by Gan *et al.* did reach a statistical significance, showing that patients with dementia with Lewy bodies have more WMHs than patients with Parkinson’s disease with dementia.^38^ Previously, the review on dementia with Lewy bodies and Parkinson’s disease with dementia by Hijazi *et al.*^2^ had highlighted inconclusive results with regards WMHs in dementia with Lewy bodies and Parkinson’s disease with dementia, potentially due to differing methods and moderate sample sizes (from 17 to 42 participants for dementia with Lewy bodies and from 20 to 88 participants for Parkinson’s disease with dementia). Therefore, our study and the study by Gan *et al*.^38^ contribute to clarify that discussion by suggesting that in large cohorts, dementia with Lewy bodies patients seem to have more WMHs than in Parkinson’s disease with dementia patients. Altogether, the current evidence suggests that patients with dementia with Lewy bodies have more WMHs than cognitively unimpaired individuals, and patients with mild cognitive impairment, Alzheimer’s disease or Parkinson’s disease with dementia and less WMHs than patients with vascular dementia, but similar levels as in mixed dementia.

MRI was used in the diagnostic work up for our participants. This could have partly explained the finding on less WMHs in dementia with Lewy bodies than in vascular dementia, but we do not expect any risk for circularity or bias in our findings for dementia with Lewy bodies versus the diagnostic groups of cognitively unimpaired, mild cognitive impairment, Alzheimer’s disease and Parkinson’s disease with dementia. In terms of biological mechanisms, WMHs are usually presumed to be of vascular origin,^8^ but they may also be associated with neurodegenerative processes beyond vascular aetiology, at least in Alzheimer’s disease.^39,40^ As such we encourage future WMH studies comparing multiple diagnostic groups, helping to fully understand differences and similarities between the different types of dementia, as the field is very limited at the moment. Increasing our understanding of the pathogenesis behind WMHs will ultimately inform on their clinical use, for example with implications for treatment decisions. That understanding can also have implications for the differential diagnosis of dementia, moving the field forward to acknowledge mixed forms of dementia beyond the well-established Alzheimer’s disease plus vascular dementia mixed dementia form.

In terms of the association of WMHs with MTA, we found that patients with dementia with Lewy bodies with WMHs had greater atrophy in the medial temporal lobe than dementia with Lewy bodies patients without WMHs. To our knowledge, only Joki *et al.*^26^ also investigated the association between WMH and MTA using visual rating scales as in our study. The authors found that periventricular hyperintensities were associated with MTA in dementia with Lewy bodies, but deep and subcortical hyperintensities were not associated with MTA. Hence, the association between WMHs and atrophy could be regional rather than global. In this line, three recent studies investigated regional measures of atrophy and semi or fully automated methods for global WMH. The measures of atrophy included brain areas that overlap only partially with the areas assessed by the MTA rating scale used in our study.^9,19,41^ In Ferreira *et al*.^19^ we found no statistically significant association between global WMHs and hippocampal volume in dementia with Lewy bodies. However, a significant association was observed for fusiform volume.^19^ The fusiform gyrus is adjacent to medial temporal regions included in the MTA scale and atrophy in fusiform may thus contribute to width of temporal horn and choroid fissure, which are captured by the MTA scale. When assessing cortical thickness instead of volume, we could not find any statistically significant association for global WMHs with fusiform thickness in another dementia with Lewy bodies study.^9^ We did not either find significant associations with thickness in other regions covered by the MTA scale or hippocampal volume.^9^ The different results could be due to different sensitivities of the MRI methods used across these studies, but they could also partly be cohort specific. In this line, these findings suggest that regional vulnerabilities could be expressed differently across dementia with Lewy bodies patients, who may be represented differently across cohorts. Indeed, in a recent publication, we showed three distinct patterns of atrophy in dementia with Lewy bodies. Only a subgroup of dementia with Lewy bodies patients with more widespread atrophy including atrophy in medial temporal lobes, had a higher WMH volume.^41^ Taking all these findings together, it is likely that WMHs are associated with atrophy in medial temporal areas in dementia with Lewy bodies, but the mechanism underlying this association should be further investigated, ideally in studies including regional measures not only of both WMHs and atrophy.

Indeed, our models for WMHs and Alzheimer’s disease biomarkers predicting MTA could shed some light on the potential mechanism for WMHs and MTA. We demonstrated that WMHs were no longer significantly associated with MTA in dementia with Lewy bodies when Alzheimer’s disease biomarkers were also in the model. Instead, patients with dementia with Lewy bodies and a positive Aβ biomarker showed more MTA. The medial temporal lobe is often spared in dementia with Lewy bodies and is used as a supportive biomarker in the diagnosis of dementia with Lewy bodies.^1^ For example, the absence of MTA *in vivo* can distinguish pathologically confirmed dementia with Lewy bodies from Alzheimer’s disease.^15,16^ However, not all patients with dementia with Lewy bodies have preserved medial temporal lobes. Patients with dementia with Lewy bodies and a positive Aβ biomarker had more MTA than dementia with Lewy bodies patients with a negative Alzheimer’s disease biomarker in a clinical sample,^17^ and smaller hippocampus in a pathologically confirmed sample.^18^ What it is not fully understood yet is the interplay between Aβ and WMHs in predicting MTA. A first attempt to answer that question is the study by Abdelnour *et al.*,^42^ using E-DLB data. The authors identified subgroups of patients with dementia with Lewy bodies based on Alzheimer’s disease biomarkers, lobar atrophy and clinical features and reported WMHs across subgroups. They found that patients with dementia with Lewy bodies with Aβ positivity and more MTA had more WMHs.^42^ However, to our knowledge, only our current study and the recent article by Ferreira *et al.*^19^ have explicitly modelled the interplay between Aβ and WMHs in predicting MTA in dementia with Lewy bodies. Using visual rating scales and CSF biomarkers, our findings suggest that whilst WMHs are associated with MTA, that association may partly depend on Aβ status. Using research-oriented MRI and PET biomarkers, Ferreira *et al.*^19^ showed that the volume of the fusiform gyrus could be predicted by a double mechanism including one path for global WMHs and one separate path for regional tau via regional Aβ.^19^ The visual ratings and CSF biomarkers in our study are clinically available but lack the spatial granularity of MRI and PET measures in Ferreira *et al*.^19^ These findings combined suggest that there is a complex interplay between WMHs, Alzheimer’s disease biomarkers and MTA, whilst the exact biological mechanisms are not yet understood and require future investigation with both regional and global biomarker measures.

We found no statistically significant association between WMHs and MMSE in dementia with Lewy bodies. We previously showed that more WMHs had only a modest association with worse MMSE scores in dementia with Lewy bodies, using a different method for WMHs in a smaller sample minimally overlapped with our current sample.^9^ The association of WMHs with cognition in dementia with Lewy bodies has been previously discussed in very few studies.^2^ Chen *et al.*^10^ did find an association between more WMHs and cognitive impairment in dementia with Lewy bodies using a different cognitive test, i.e. MoCA. The MoCA test includes executive components to a larger extent than the MMSE test used in our study. In Alzheimer’s disease, WMHs are more associated with executive function than with memory.^43^ Therefore, MMSE may be less sensitive to WMH-related cognitive impairment than MoCA. Further, participants in Chen *et al.*^10^ were at a more severe cognitive stage and had less years of education than in our cohort, which could provide more variance in the data and thus explain the different results of our study.

To summarize, WMHs were common in dementia with Lewy bodies and were associated with MTA. However, when Alzheimer’s disease biomarkers were added to the model, WMHs were no longer statistically significantly associated with MTA, whilst a positive Aβ biomarker significantly predicted more MTA. WMHs were not associated with MMSE in our cohort. Our second main goal in this study was to elucidate whether these associations were dementia with Lewy bodies specific, throughout testing for statistical interactions between biomarkers and the diagnostic group in our models. We demonstrated that for the association of WMHs with MTA, findings in the group of patients with dementia with Lewy bodies differed from those in the cognitively unimpaired and mixed dementia groups. Among patients with dementia with Lewy bodies, the association between WMHs and MTA was statistically stronger than in both the groups of cognitively unimpaired and patients with mixed dementia. We observed that in the cognitively unimpaired group, WMHs and MTA levels were low whilst in the mixed dementia group, WMHs and MTA levels were high, largely independently of WMH status. This suggests that MTA in dementia with Lewy bodies is partly influenced by WMH, whilst this does not seem to be the case among cognitively unimpaired and in mixed dementia, highlighting the specificity of this finding in dementia with Lewy bodies. We then evaluated the interaction between Aβ and the diagnostic group in the prediction of MTA. We demonstrated that patients with dementia with Lewy bodies with a positive Aβ biomarker had more MTA than patients with a negative Aβ biomarker, whilst MTA scores were independent of Aβ status in the group of patients with mild cognitive impairment. This interaction highlights the association between Aβ and MTA in dementia with Lewy bodies and may indicate different mechanisms of neurodegeneration than in mild cognitive impairment. Finally, the interaction for MMSE showed that whilst WMHs were not associated with MMSE in dementia with Lewy bodies, Alzheimer’s disease patients with more WMHs had worse MMSE scores. Despite these statistically significant interactions, we note that the other groups in the models (vascular dementia, Parkinson’s disease with dementia) did not show any statistical interaction with dementia with Lewy bodies. Overall, this finding would suggest that the effect of WMHs is rather similar across diagnoses, but their load, regional placement and interplay with alpha-synuclein and Alzheimer’s disease-related pathological changes may differ. For example, whilst it is still debated whether there is a difference in the frequency of WMHs in dementia with Lewy bodies and Parkinson’s disease with dementia, it has been suggested that the regional distribution of WMHs does differ.^2^ Similarly, differences in the regional distribution of WMHs have also been reported between dementia with Lewy bodies and Alzheimer’s disease.^2^ Our study is a first step in understanding the interplay between WMHs, Alzheimer’s disease biomarkers, MTA and MMSE. Future studies should evaluate these factors with methods that have high granularity, for example regional measures for WMHs or cognitive tests that assess specific cognitive domains in depth. Using cognitive tests with higher granularity would expand our current findings in several ways. First, tests for global cognitive screening like MMSE may have limited ability to detect milder forms of cognitive impairment, whilst detailed neuropsychological tests would better characterize cognitive profiles specific to different types of dementia and mild cognitive impairment. Second, assessing cognition with detailed neuropsychological tests would allow for an increased understanding of the relationship and synergies of pathologies such as WMHs and Alzheimer’s disease biomarkers with cognition. Further, the inclusion of longitudinal data would also be important in understanding the sequence of cognitive impairments in relation to pathological and clinical trajectories across diagnoses.

This study has some limitations. First, we used data from four cohorts, where one was a multi-centre study across Europe, one was a naturalistic multi-centre study including specialist clinics, one was a research-oriented specialized clinic and one was population-based. Although this approach increases representativeness and generalization of results, it may also introduce some variability in terms of measures and diagnostic procedures across cohorts, despite our efforts to harmonize the data for statistical analysis. To partly address this challenge, we ensured that diagnostic procedures were comparable and visual ratings were performed following established clinical guidelines across all cohorts. Second, diagnoses were primarily based on clinical judgement and lacked neuropathological confirmation. Previous studies have shown varied concordance between clinical and neuropathological diagnosis,^44,45^ which should be considered when interpreting our findings. Third, although dementia with Lewy bodies and Parkinson’s disease with dementia are often discussed to be part of the same spectrum of alpha-synuclein-related pathology, both are common clinical diagnoses in clinical settings and the cohorts used in this study. Therefore, we aimed to include both groups in our analyses and compare them to further clarify any potential differences in terms of WMHs, MTA, CSF biomarkers and MMSE. The clinics had the information about the patients’ onset of cognitive and motor symptoms for implementation of the 1-year rule for the differential diagnosis between dementia with Lewy bodies and Parkinson’s disease with dementia.^1^ However, we could not access that specific data for reporting and analysis in our current study. Fourth, we used Fazekas to assess WMHs. Whilst Fazekas is a clinically available and easy-to-use scale, it only reflects one aspect of cerebrovascular pathology, whilst there are imaging measures for the assessment of other aspects such as cerebral microbleeds or lacunes.^8^ Additionally, Fazekas does not provide the quantitative volume of WMHs nor detailed information on regional placement. In this line, three recent imaging studies characterized the regional pattern of cholinergic alterations in grey and white matter in dementia with Lewy bodies,^46-48^ and we demonstrated an association between WMHs and atrophy in brain areas that receive prominent cholinergic input, in dementia with Lewy bodies.^9^ Hence, an interesting prospect for the future would be to evaluate the regional placement of WMHs in areas associated with the cholinergic system in dementia with Lewy bodies. The Fazekas scale lacks granularity compared to automated volumetric methods for WMHs. However, Fazekas is less sensitive to variability in MRI scanners and processing methods common in multi-centre and multi-cohort studies, which guided method choice in our study. Finally, our sample was smaller for CSF analyses than for the main analysis, and it was younger and had less WMHs. This could in part reflect clinical decisions and the lower referral rate for lumbar punctures in older patients with more comorbidities. At the same time, this could have reduced our possibilities to capture stronger associations of WMHs with Alzheimer’s disease biomarkers and atrophy, since all these increase with age in dementia with Lewy bodies.^9,12,49^

In conclusion, this study demonstrates that WMHs are more frequent in dementia with Lewy bodies than in cognitively unimpaired, mild cognitive impairment and Alzheimer’s disease, but less frequent than in vascular dementia. In dementia with Lewy bodies, WMHs were associated with MTA, but this association could depend on Aβ positivity. We also observed several statistical interactions indicating partly dementia with Lewy body-specific results. The interactions overall suggest stronger associations in dementia with Lewy bodies in measures reflecting biological mechanisms (WMHs, Aβ and MTA), which do not seem to translate to stronger associations in global cognitive performance assessed with MMSE. We believe these results reflect the added contribution of cerebrovascular and Aβ co-pathologies to dementia with Lewy body pathogenesis. Whilst the biological contributions of WMHs may be similar across diagnoses, their effect may depend on the presence of co-pathologies, which in dementia with Lewy bodies have larger variability than in cognitively unimpaired, mild cognitive impairment and mixed dementia (the groups we captured biological interactions with). To advance the current field, it will be important to continue investigating the influence of these pathologies across multiple dementia diagnoses and their prodromal stages, elucidating potentially shared mechanisms but also distinct contributions to clinical presentations.

## Supplementary Material

fcae290_Supplementary_Data

## Data Availability

The data may be available upon reasonable request if legal and ethical requirements can be met. Included cohorts operate independently regarding data sharing and the corresponding authors can facilitate contact with each cohort.
