# Supplementary material for: Cerebrovascular and Alzheimer’s disease biomarkers in dementia with Lewy bodies and other dementias
Source: Brain Commun. 2024 Aug 28;6(5):fcae290. doi: 10.1093/braincomms/fcae290 (PMC11406466; doi:10.1093/braincomms/fcae290)
Supplement: fcae290_Supplementary_Data [file fcae290_supplementary_data.docx]

**Supplementary Table 1: Participants from each cohort and total participants with available CSF and CT (otherwise MRI available)**

| **Diagnosis** | **E-DLB** | **H70** | **KIDS** | **MemClin** | **Total** | **CSF**  **available** | **CT**  **available**  **(all from MemClin)** |
| --- | --- | --- | --- | --- | --- | --- | --- |
| Dementia with Lewy bodies | 283 (91) | - | - | 28 (9) | 311 | 84 (27) | 10 (3) |
| Cognitively unimpaired | 23 (2) | 774 (51) | 373 (25) | 335 (22) | 1505 | 716 (48) | 173 (11) |
| Mild Cognitive impairment | - | - | 427 (29) | 1062 (71) | 1489 | 784 (53) | 723 (49) |
| Alzheimer’s disease | 150 (21) | - | 322 (46) | 236 (33) | 708 | 362 (51) | 170 (24) |
| Mixed dementia | - | - | 108 (40) | 160 (60) | 268 | 156 (58) | 109 (41) |
| Vascular dementia | - | - | 60 (41) | 88 (59) | 148 | 61 (41) | 67 (45) |
| Parkinson’s disease dementia | 90 (75) | - | 22 (18) | 8 (7) | 120 | 28 (23) | 6 (5) |
| Total | 546 (12) | 774 (17) | 1312 (29) | 1917 (42) | 4549 | 2191 (48) | 1258 (28) |

Data is reported as count (%). CT was only used in the MemClin-cohort.

**Supplementary table 2: Overview of harmonized diagnostic criteria across cohorts**

| **Diagnosis** | **E-DLB** | **H70** | **KIDS** | **MemClin** |
| --- | --- | --- | --- | --- |
| Dementia with Lewy bodies | ICD-10 code  F02.8, G31.8A | - | - | ICD-10 code  F02.8, G31.8A |
| Cognitively unimpaired | Recruited controls | Population based controls | Z03.2A, Z03.3 and R41.8A | Z03.2A, Z03.3  and R41.8A |
| Mild Cognitive impairment | - | - | ICD-10 code F067 | ICD-10 code  F067 |
| Alzheimer’s disease | ICD-10 code  F00.0/F00.1 | - | ICD-10 code  F00.0/F00.1 | ICD-10 code  F00.0/F00.1 |
| Mixed dementia | - | - | ICD-10 code F00.2/F00.9 | ICD-10 code  F00.2 |
| Vascular dementia | - | - | ICD-10 code  F01.1, F01.2, F01.3, F01.9  and CADASIL | ICD-10 code  F01.1, F01.2,  F01.3 and F01.9 |
| Parkinson’s disease dementia | ICD-10 code  F02.3 | - | ICD-10 code  F02.3 | ICD-10 code  F02.3 |

More detailed information about recruitment and diagnostic/inclusion/exclusion criteria are available in each original publication:

E-DLB^1^ DLB diagnosis based upon the third consensus criteria.^2^ H70^3^, KIDS^4^, MemClin^5^

**Supplementary table 3: Overview of CSF cut-offs**

| **Cohort** | **Type** | **Method** |
| --- | --- | --- |
| E-DLB^6,7^ | Center-specific cut-offs | Enzyme-linked immunosorbent assay (ELISA) and Biosource Europe S.A  Cut-offs:  Ljubljana: <550 pg/mL for Aβ42, >80 pg/mL for ptau  Stockholm: <550 pg/mL for Aβ42, >80 pg/mL for ptau  Stavanger: <482 pg/mL for Aβ42, >52 pg/mL for ptau  Strasbourg: <500 pg/mL for Aβ42, >60 pg/mL for ptau  Prague: <582pg/mL for Aβ42, >57 pg/mL for ptau  Amsterdam: <550 pg/mL for Aβ42, >52 pg/mL for ptau |
| H70^8^ |  | INNOTEST Aβ1-42 and INNOTEST htau Ag  and PHOSPHO_TAU [181P], Fujirebio  Cutoff ≤530 pg/mL for Aβ42, ≥80 pg/mL for ptau |
| KIDS^9^ | Procedure and cut-off used in clinical routine | Enzyme-linked immunosorbent assay (ELISA)  ≤550 pg/mL for Aβ42, ≥80 pg/mL for ptau. |
| MemClin^5^ | Procedure and cut-off used in clinical routine | Enzyme-linked immunosorbent assay (ELISA),  Electrochemiluminiscence Immuno Assay (ECLIA), Chemiluminescence enzyme immunoassay (CLEIA).  ELISA:  ≤550 pg/mL for Aβ42, ≥80 pg/mL for ptau.  ECLIA:  ≤599 pg/mL for Aβ42, ≥56,5 pg/mL for ptau.  CLEIA:  ≤620 pg/mL for Aβ42, ≥61 pg/mL for ptau |

**Supplementary Table 4: Patients with DLB divided into high and low groups on WMH, MTA and Aβ.**

| **Diagnosis** | **High WMH Score** | **Low WMH Score** | **High MTA Score** | **Low MTA Score** | **Aβ, High** | **Aβ, Low** |
| --- | --- | --- | --- | --- | --- | --- |
| N | 135 | 176 | 125 | 134 | 28 | 56 |
| Age, years | 77.2 (6.4) | 70.5 (8.1) | 75.5 (5.9) | 70.8 (9.4) | 71.8 (7.0) | 69.4 (7.7) |
| Women, N (%) | 59 (44) | 60 (34) | 51 (41) | 48 (39) | 8 (29) | 8 (14) |
| Education, years | 10.8 (3.9) | 11.2 (4.0) | 10.8 (4.0) | 11.1 (4.0) | 11.3 (3.2) | 12.7 (4.3) |
| MMSE, total score | 22.5 (3.9) | 22.7 (4.1) | 22.4 (4.1) | 22.9 (3.9) | 22.8 (4.4) | 23.8 (3.3) |
| High WMH Score N (%) | 135 (100) | 0 (0) | 70 (56) | 42 (31) | 12 (43) | 17 (30) |
| MTA, High score, N (%) | 70 (62) | 55 (37) | 125 (100) | 0 (0) | 19 (70) | 20 (36) |
| Aβ, High, N (%) | 12 (41) | 16 (29) | 19 (49) | 8 (18) | 28 (100) | 0 |
| P-tau, High, N (%) | 13 (45) | 18 (33) | 12 (31) | 18 (41) | 11 (39) | 20 (36) |

Available data: 311 participants with DLB, MTA rating available for 259 and CSF for 84.

References:

1. Oppedal K, Borda MG, Ferreira D, Westman E, Aarsland D. European DLB consortium: diagnostic and prognostic biomarkers in dementia with Lewy bodies, a multicenter international initiative. *Neurodegenerative disease management*. 2019;9(5):247-250. doi:10.2217/nmt-2019-0016

2. McKeith IG, Dickson DW, Lowe J*, et al*. Diagnosis and management of dementia with Lewy bodies: Third report of the DLB consortium. *Neurology*. 2005;65(12):1863-1872. doi:10.1212/01.wnl.0000187889.17253.b1

3. Rydberg Sterner T, Ahlner F, Blennow K*, et al*. The Gothenburg H70 Birth cohort study 2014–16: design, methods and study population. *European journal of epidemiology*. 2019;34(2):191-209. doi:10.1007/s10654-018-0459-8

4. Shams S, Martola J, Charidimou A*, et al*. Topography and Determinants of Magnetic Resonance Imaging (MRI)‐Visible Perivascular Spaces in a Large Memory Clinic Cohort. *Journal of the American Heart Association*. 2017;6(9):n/a. doi:10.1161/JAHA.117.006279

5. Ekman U, Ferreira D, Muehlboeck JS*, et al*. The MemClin project: a prospective multi memory clinics study targeting early stages of cognitive impairment. *BMC geriatrics*. 2020;20(1):93. doi:10.1186/s12877-020-1478-3

6. Ferreira D, Przybelski SA, Lesnick TG*, et al*. β-Amyloid and tau biomarkers and clinical phenotype in dementia with Lewy bodies. *Neurology*. 2020;95(24):e3257-e3268. doi:10.1212/WNL.0000000000010943

7. Van Steenoven I, Aarsland D, Weintraub D*, et al*. Cerebrospinal Fluid Alzheimer's Disease Biomarkers Across the Spectrum of Lewy Body Diseases: Results from a Large Multicenter Cohort. *Journal of Alzheimer's disease*. 2016;54(1):287-295. doi:10.3233/JAD-160322

8. Samuelsson J, Kern S, Zetterberg H*, et al*. A Western‐style dietary pattern is associated with cerebrospinal fluid biomarker levels for preclinical Alzheimer's disease—A population‐based cross‐sectional study among 70‐year‐olds. *Alzheimer's & dementia : translational research & clinical interventions*. 2021;7(1):e12183-n/a. doi:10.1002/trc2.12183

9. Ferreira D, Shams S, Cavallin L*, et al*. The contribution of small vessel disease to subtypes of Alzheimer's disease: a study on cerebrospinal fluid and imaging biomarkers. *Neurobiology of aging*. 2018;70:18-29. doi:10.1016/j.neurobiolaging.2018.05.028
